# A retrospective study of the relationship between the pathologic subtype and lymph node metastasis of lung adenocarcinomas of ≤3 cm diameter

**DOI:** 10.1097/MD.0000000000021453

**Published:** 2020-09-04

**Authors:** Wenwei Lin, Mingcheng Huang, Zhenyang Zhang, Tianci Chai, Sui Chen, Lei Gao, Jiangbo Lin, Mingqiang Kang

**Affiliations:** a Department of Thoracic Surgery, The Union Clinical Medical College of Fujian Medical University, Fuzhou; b Department of Thoracic Surgery, Fujian Medical Hospital Affiliated to Zhangzhou Hospital, Zhangzhou, Fujian Province, China.

**Keywords:** diameter, lung adenocarcinoma, lymph node metastasis, pathology

## Abstract

To analyze the relationship between pathologic subtype and lymph node metastasis for lung adenocarcinomas of ≤3 cm diameter.

We retrospectively studied 384 patients with operable lung adenocarcinomas of ≤3 cm diameter that had been radically resected by lobectomy or anatomic segmentectomy with systematic nodal dissection, at the Fujian Medical University Union Hospital between March 2014 and March 2016.

Lymph node metastasis pN1 + pN2 (pN+) was found in 2 of 104 (1.9%) patients with tumor diameter ≤1.0 cm, 12 of 159 (7.5%) patients with tumor diameter >1.0 cm but ≤2.0 cm, and 35 of 121 (28.9%) patients with tumor size >2.0 cm but ≤3.0 cm (*P *< .01). Lymph node metastasis pN+ was found in 19 of 53 (35.8%) patients with visceral invasion pleural (VIP) and 30 of 331 (9.0%) patients without VIP (*P *< .05). It was also found in 16 of 51 (31.3%) patients with high serum CEA concentrations and 28 of 297 (9.4%) patients with normal concentrations (*P *< .05). In a multivariate analysis, tumor diameter, VIP, high serum CEA concentration, and pathologic subtype were significant risk factors. The prevalences of lymph node metastasis pN+ were: 0.0% (0/2), 0.0% (0/89), 3.2% (1/31), 16.2% (34/209), 7.7% (1/13), 46.7% (7/15), 100% (4/4), and 11.8% (2/17) for adenocarcinoma in situ (AIS); minimally invasive adenocarcinoma (MIA); predominantly lepidic (LEP), acinar (ACI), papillary, solid (SOL), and micropapillary (MIP) tumors; and variants of invasive adenocarcinoma, respectively (*P *< .05). For predominant SOL and MIP tumors, the prevalences of lymph node involvement were significantly higher than for the other subtypes.

We have shown that lymph node metastasis in patients with tumor diameter ≤3 cm differs according to lung adenocarcinoma subtype. AIS and MIA were not associated with lymph node metastasis; therefore, systematic nodal dissection may be unnecessary. The prevalence of lymph node metastasis rate was low for LEP, suggesting that systemic lymph node sampling is sufficient. In contrast, for other pathologic subtypes, including SOL and MIP, systematic lymph node dissection should be performed.

## 1 Introduction

Lung cancer is a serious threat to health, and its incidence and mortality rate are the highest of all the malignant tumors in China.^[1–3]^ Adenocarcinoma is currently the most common pathologic type of nonsmall cell lung cancer (NSCLC).^[4]^ In general, pulmonary lobectomy is regarded as the gold standard therapy for lung cancer. However, in recent years, studies have found that early sublobectomy can be equally as effective as lobectomy and is associated with superior retention of lung function, which is important for patient quality of life. Sublobectomy is performed in patients who do not have lymph node metastasis and in tumors containing “ground-glass nodules” in particular. The identification of such patients, who will avoid excessive treatment, is an important component of individualized treatment. Because early lung cancer lesions are small, and preoperative biopsy can cause the dissemination of tumor cells, it is crucial to identify the presence of lymph node metastasis to determine the most appropriate surgical approach.

TNM stage is the most important prognostic indicator for lung cancer, whereas traditional pathologic classification is not related to the treatment or prognosis. Also, many retrospective studies have shown that the classification of lung adenocarcinoma published by the IASLC/ATS/ERS in 2011 is useful for the prediction of mortality and recurrence.^[5–8]^ With the development and popularization of imaging technologies, such as the wide application of low-dose spiral computed tomography, high-resolution computed tomography (CT), thin-layer scanning, and 3-dimensional reconstruction technologies, increasing numbers of small lung cancers with tumor diameter ≤3 cm are being detected in the clinic. However, lymph node metastasis is not easily identified prior to surgery if the primary tumor has a diameter ≤3 cm, even using thin-layer CT, PET-CT, or other modalities. In addition, it is unclear whether the pathologic subtype of lung adenocarcinoma, especially of those tumors with a diameter ≤3 cm, affects the likelihood of lymph node metastasis.

We aimed to determine the relationship between pathologic subtype and lymph node metastasis in patients undergoing radical resection of lung adenocarcinomas of ≤3 cm diameter by retrospective analysis of the cases enrolled at our center and to identify the risk factors affecting the likelihood of metastasis. The findings provide a basis for the likelihood of lymph node metastasis to guide lung cancer surgery.

## 2 Materials and methods

### 2.1 Baseline data

We identified 384 patients (152 men and 232 women) who had received a pathological diagnosis of lung adenocarcinoma of ≤3 cm diameter and had undergone radical resection of the tumor, by lobectomy or anatomic segmentectomy plus systematic nodal dissection, under thoracoscopic guidance at the Fujian Medical University Union Hospital between March 2014 and March 2016. The inclusion criteria were radical surgery for lung cancer (anatomical lobectomy or segmentectomy plus systemic lymph node dissection), confirmation of lung adenocarcinoma by postoperative pathology, classification of the disease according to the lung adenocarcinoma histologic classification published by IASLC/ATS/ERS in 2011, and tumor diameter ≤3 cm. The exclusion criteria were preoperative chemotherapy or radiation therapy and a previous history of cancer. The basic clinical data and related characteristics are shown in Table 1. This study was approved by the Ethics Committee of the Fujian Medical University Union Hospital. All the participants gave their written informed consent prior to their inclusion in the study.

**Table 1:**
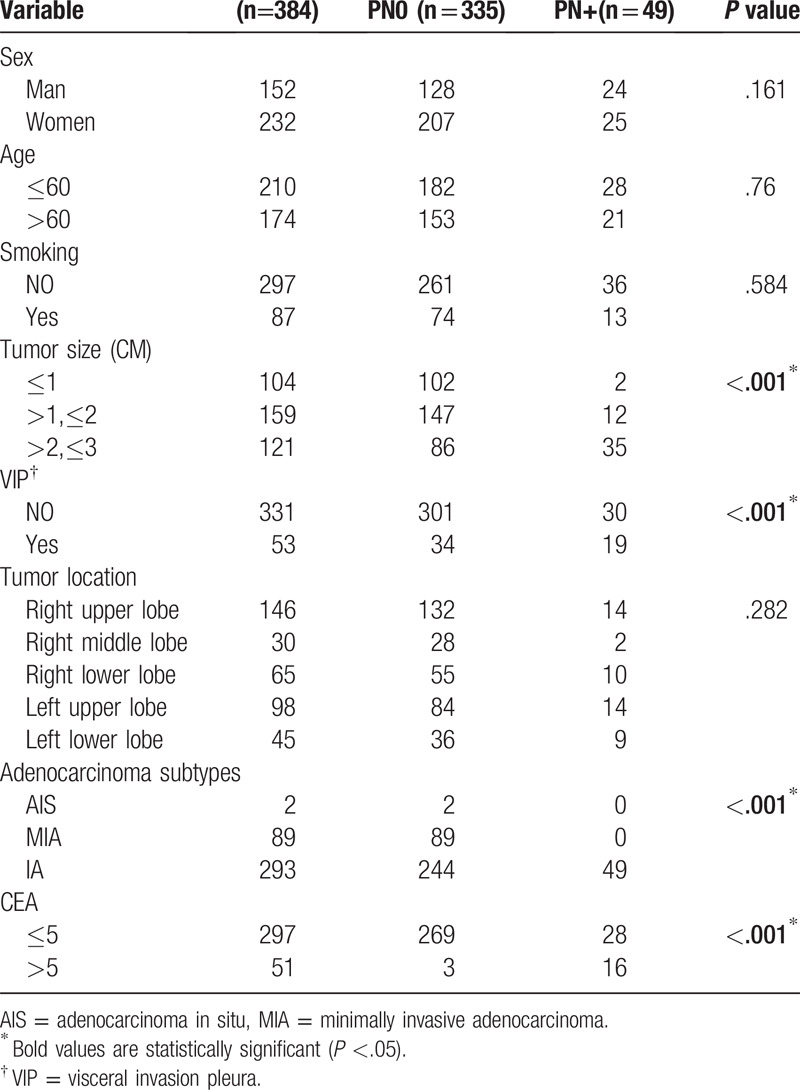
Patient characteristics.

### 2.2 Surgical treatment

All the participants underwent radical resection under thoracoscopic guidance for lung cancer after comprehensive preoperative assessment of their general condition and neoplastic disease. Each underwent surgery under general anesthesia with a double-lumen endotracheal intubation and in a lateral position on the operating table. Three hundred thirty-four patients underwent lobectomy and 50 underwent anatomic segmentectomy plus systemic lymph node dissection. The dissected lymph nodes were clearly marked and recorded.

### 2.3 Statistics

All the data were analyzed using SPSS v.19.0 (IBM, Inc, Armonk, NY). The relationships between clinical data, pathologic features, and lymph node metastasis for patients with lung adenocarcinomas of ≤3 cm diameter were analyzed using *χ*^2^ and Fisher exact tests. Multivariate analysis was performed using logistic regression analysis to determine the prognosis for these patients. *P* < .05 was considered to represent statistical significance.

## 3 Results

### 3.1 Relationships between clinical data, pathologic features, and lymph node metastasis

A total of 384 patients met the above criteria and were included in the study. Of these, 87% (335/384) did not have lymph node metastasis, whereas 12.8% (49/384) of them did pN+. Of the patients, 5.2% were pN1 (20/384) and 7.6% were pN2 (29/384) (*P* < .05) (Table 1). The prevalences of lymph node metastasis pN+ were 1.9% (2/104), 7.5% (12/159), and 28.9% (35/121) in patients with tumor diameter (d) ≤1.0 cm, 1.0 cm < d ≤ 2.0 cm, and 2.0 cm < d ≤ 3.0 cm, respectively (*P* < .05) (Table 1). Patients with visceral invasion pleura (VIP) and those without had prevalences of lymph node metastasis pN+ of 35.8% (19/53) and 9.0% (30/331), respectively (*P* < .05) (Table 1). The prevalences of lymph node metastasis pN+ were 31.3% (16/51) and 9.4% (28/297) in patients with high (>5 ng/mL) and normal blood CEA concentration, respectively. When the lung adenocarcinomas were classified as adenocarcinoma in situ (AIS), minimally invasive adenocarcinoma (MIA), or IA, the prevalences of lymph node metastasis (pN1 + pN2) were 0.0% (0/2), 0.0% (0/89), and 16.7% (49/293), respectively (*P* < .05) (Table 1). However, sex, age, smoking history, and the location of the tumor were not related to lymph node metastasis (*P* > .05) (Table 1).

### 3.2 Multivariate analysis of lymph node metastasis associated with lung adenocarcinomas of ≤3 cm diameter

Multivariate analysis of the factors potentially associated with lymph node metastasis (age, sex, smoking history, tumor location, tumor diameter, visceral pleural involvement, pathologic subtype, and serum CEA concentration) showed that tumor diameter, VIP, pathologic subtype, and high serum CEA concentration predicted lymph node metastasis (*P* < .05) (Table 2).

**Table 2:**
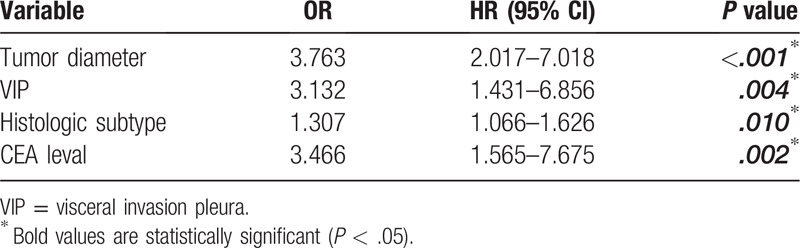
Multivariate analysis of lymph node metastasis with adenocarcinoma with a tumor diameter ≤ 3 cm.

### 3.3 The prevalences of the pathologic subtypes of lung adenocarcinoma vary with tumor size

**Figure 1:**
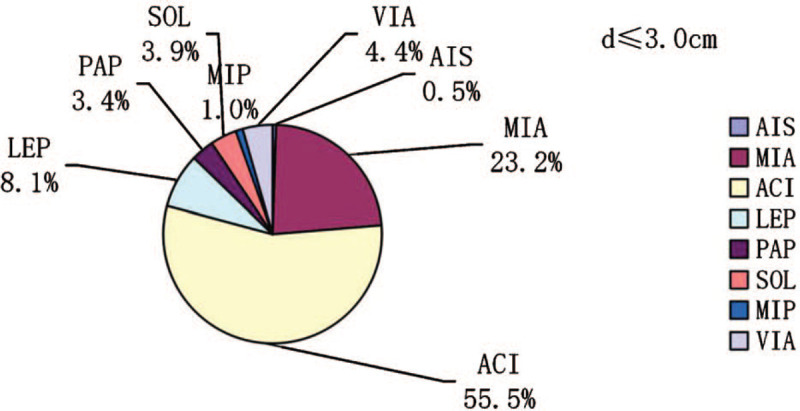
The prevalences of the pathologic subtypes in lung adenocarcinomas with diameters ≤3.0 cm.

**Figure 2:**
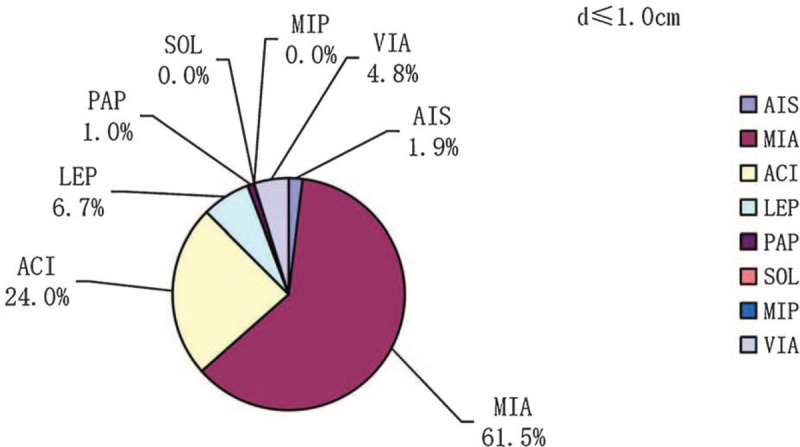
The prevalences of the pathologic subtypes in lung adenocarcinomas with diameters ≤1.0 cm.

**Figure 3:**
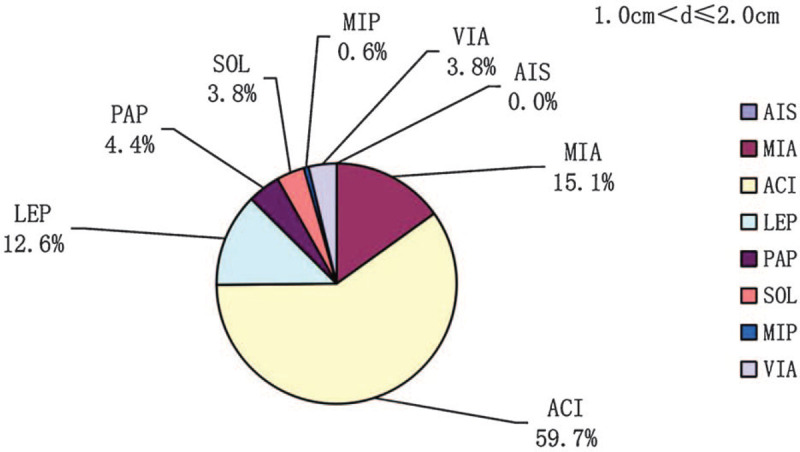
The prevalences of the pathologic subtypes in lung adenocarcinomas with diameters 1 cm < d ≤ 2 cm. d = diameter.

**Figure 4:**
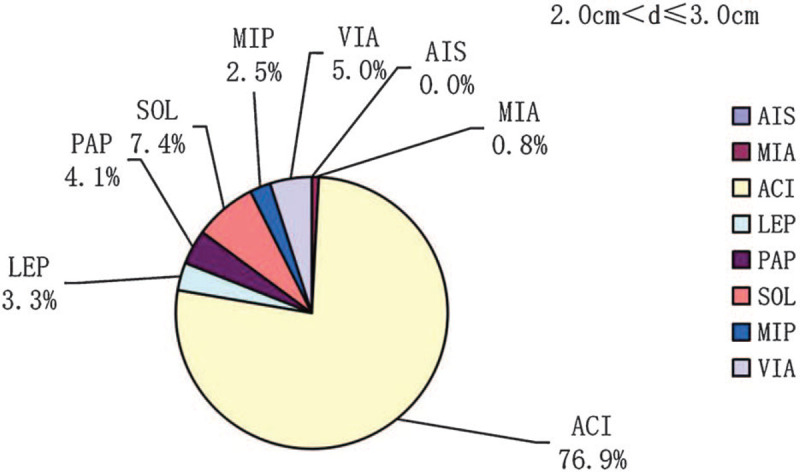
The prevalences of the pathologic subtypes in lung adenocarcinomas with diameters 2 cm < d ≤ 3 cm. d = diameter.

In participants with tumor diameter ≤3.0 cm, the prevalences of each lung adenocarcinoma subtype (AIS, MIA, acinar [ACI], lepidic [LEP], papillary [PAP], solid [SOL], and micropapillary [MIP]) and the variant of invasive adenocarcinoma (VIA) were 0.5%, 23.2%, 55.5%, 8.1%, 3.4%, 3.9%, 1%, and 4.4%, respectively (Fig. 1). The prevalences of ACI, SOL, and MIP increased with increasing tumor diameter and were 24.0%, 0.0%, and 0.0%; 59.7%, 3.8%, and 0.6%; and 76.9%, 7.4%, and 2.5% in participants with d ≤ 1.0 cm, 1.0 cm < d ≤ 2.0 cm, and 2.0 cm < d ≤ 3. 0 cm, respectively (Figs. 2–4). In contrast, the prevalences of AIS and MIA decreased as the diameter of the tumor increased and were 1.9% and 61.5%, 0.0%, and 15.1%, and 0.0% and 0.8% in d ≤ 1.0 cm, 1.0 cm < d ≤ 2.0 cm, and 2.0 cm < d ≤ 3.0 cm tumors, respectively (Figs. 2–4).

### 3.4 Relationship between pathologic subtype of lung adenocarcinoma and lymph node metastasis

Of the 384 participants, the prevalences of lymph node metastasis pN+ in participants with pathologic subtypes classified according to the 2011 IASLC/ATS/ERS guidelines were as follows: AIS, 0.0%; MIA, 0.0%; ACI, 16.0%; LEP, 3.2%; PAP, 7.7%; SOL, 46.7%; MIP, 100%; and VIA, 12.0% (*P* < .05) (Tables 3 and 4). For AIS and MIA, there were no lymph node metastases, regardless of tumor size. Conversely, for SOL and MIP, the prevalence of lymph node metastasis was significantly higher than for other pathologic subtypes. The prevalence of lymph node metastasis associated with SOL was 50% for 1.0 cm < d ≤ 2.0 cm tumors, and those associated with SOL and MIP were 44% and 100%, respectively, for 2.0 cm < d ≤ 3.0 cm tumors.

**Table 3:**
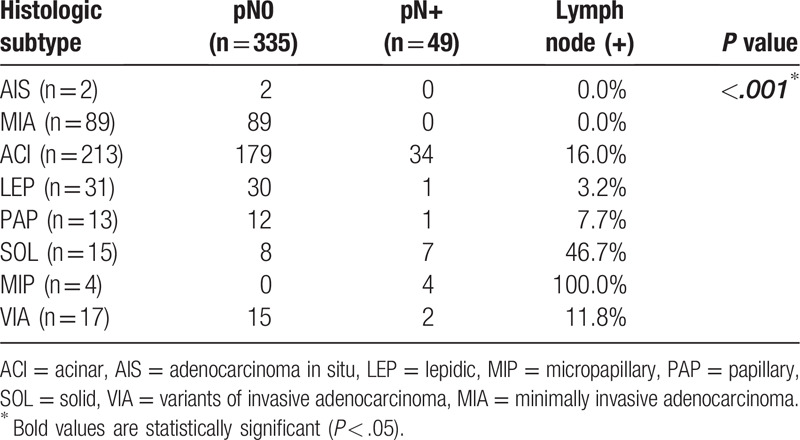
Correlation between lymph node metastasis and pathologic subtypes of lung adenocarcinoma with a tumor diameter ≤ 3 cm.

**Table 4:**
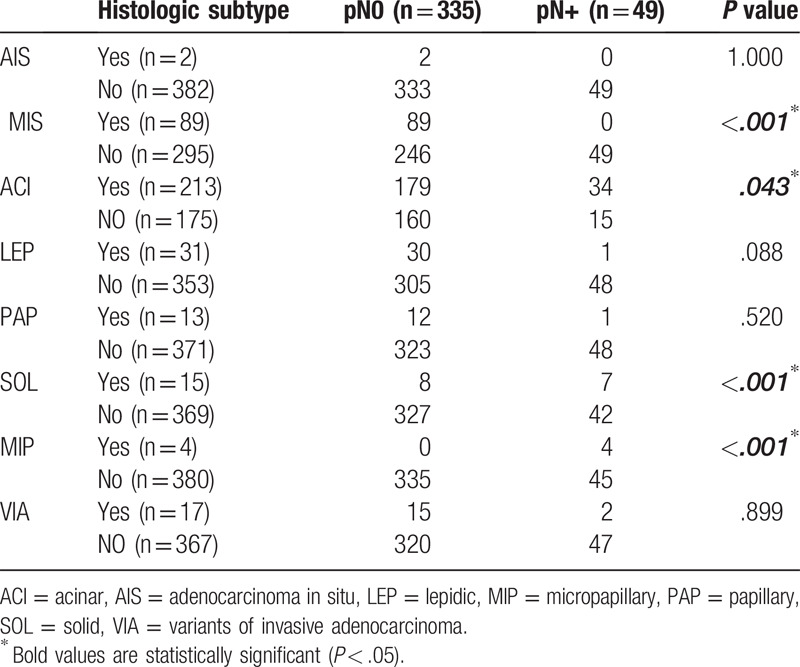
Comparison of the correlation between lymph node metastasis and pathologic subtypes of lung adenocarcinoma with a tumor diameter ≤ 3 cm.

## 4 Discussion

Lung cancer is associated with the highest overall morbidity and mortality of all tumors in Chinese people.^[1]^ With the development and popularization of CT technology and imaging diagnostics in recent years, the ability to screen for and diagnose early lung cancer has improved, and early small cell lung cancers are now more frequently treated surgically. The eighth edition of the IASLC lung adenocarcinoma TNM staging system states that tumor size has a significant effect on prognosis.^[9]^ such that as the diameter of the tumor increases, the risk of lymph node metastasis increases. However, there is still no definitive method for the accurate prediction and evaluation of lymph node metastasis prior to surgery. Surgery, including anatomical lobectomy and anatomical segmentectomy approaches, is the preferred treatment for early NSCLC, and anatomical lobectomy plus systemic lymphadenectomy is a standard surgical procedure. However, it has been shown that there are no significant differences in the prevalences of survival or recurrence between early NSCLC patients, especially with small tumors (d ≤ 2 cm) that undergo anatomical lobectomy or anatomical segmentectomy.^[10–12]^ but there is superior retention of lung function following the latter.^[13]^ Therefore, a better understanding of the lymph node metastasis associated with early lung adenocarcinomas of ≤3 cm diameter is important to guide the appropriate choice of surgical procedure. In this study, we retrospectively analyzed the relationship between lymph node metastasis and the pathologic subtype of early lung adenocarcinoma in patients treated in our center between March 2014 and March 2016.

Although the overall trend is consistent, the relationship between tumor size and lymph node metastasis shown in this study is slightly different from that shown in previous studies, which might be the result of differences in sample size. Among these previous studies, Baba et al^[14]^ analyzed patients with NSCLC with a tumor diameter ≤1 cm and reported a prevalence of lymph node metastasis of 3.4%, whereas Shi et al^[15]^ reported a prevalence in patients with a tumor diameter ≤2 cm of ∼14.1%, and Yu et al^[16]^ reported prevalences of 3.2%, 14.5%, and 31.1% in patients with tumor diameters ≤1.0 cm, >1.0 to ≤2.0 cm, and >2.0 to ≤3.0 cm, respectively. Taking these results together with our own, it seems that lymph node metastasis might occur in early lung adenocarcinomas, even if the tumor has a diameter of ≤1 cm. Therefore, it is important to more definitively determine whether systemic lymph node dissection should be performed based on the size of the primary tumor.

Several previous studies have shown that the currently recommended 2011 IASLC/ATS/ERS lung adenocarcinoma subtype classification is an effective predictor of disease prognosis in patients undergoing surgical treatment.^[8,17–21]^ Among patients who underwent complete tumor resection, those with the AIS or MIA pathologic subtypes had a better prognosis, with a lower risk of recurrence and a higher survival rate. A pooled analysis of 972 patients in 19 studies by Behera et al^[22]^ showed that the 5-year overall survival (OS) rate and the 5-year disease-free survival rate did not differ between patients with AIS and MIA. Patients with adherent-type lung adenocarcinoma have a better prognosis than those with other invasive adenocarcinoma subtypes. In contrast, patients with the MIP or SOL subtypes have higher risks of recurrence and tumor-related mortality. In most studies, patients with ACI based and PAP-type tumors, the most common subtypes in patients with lung adenocarcinoma, which together account for the majority of invasive adenocarcinoma cases, have a similar prognosis, with lower survival rates than in patients with AIS or MIA but higher than for MIP or SOL.^[23,24]^ Similarly, Yoshiya et al^[25]^ showed that for invasive lung adenocarcinomas of ≤2 cm diameter, pathologic subtype is a significant independent predictor of recurrence-free survival and suggested that adjuvant chemotherapy is required after tumor resection, even in the absence of lymph node metastasis, for patients with MIP or SOL. In this study, we found no lymph node metastasis, regardless of tumor size, for AIS and MIA tumors, and for LEP tumors, lymph node metastasis was unlikely, and even if it did occur, it was mostly pN1. However, for SOL and MIP, the prevalence of lymph node metastasis was significantly higher than that associated with other pathologic subtypes, and the lymph node stage also tended to be higher, which is consistent with the findings of previous studies.^[12,22–25]^ According to Mimae et al^[26]^ there was significantly less adherent-like growth of the tumors of patients with pathologic lymph node-positive NSCLC. It should be noted that in this study, SOL and MIP were not identified in patients with a tumor diameter ≤1.0 cm, and MIP was not identified in patients with tumors 1.0 cm < d ≤ 2.0 cm. Although it cannot be excluded that MIP tumors are always large, it is more likely that there was insufficient MIP sample size, because there were only 4 MIP cases in this study. In summary, our findings indicate that the pathologic subtype of lung adenocarcinomas with diameters ≤3.0 cm is closely related to lymph node metastasis. Specifically, AIS and MIA, and to a lesser extent LEP, tumors are less prone to lymph node metastasis, whereas SOL and MIP tumors are associated with a higher prevalence of lymph node metastasis. The association reported here between pathologic subtype of lung adenocarcinoma and lymph node metastasis is consistent with the association shown between lung adenocarcinoma subtype and prognosis in previous studies.

It is unclear whether the differing tendencies for lymph node metastasis associated with lung adenocarcinoma pathologic subtypes also affect prognosis. However, the existence of differences in prognosis associated with different lung adenocarcinoma pathologic subtypes implies that there may be differences in invasiveness between the subtypes.

Although it has not become firmly established as a predictor of lymph node metastasis, the pathologic subtype of lung adenocarcinoma, especially in tumors with diameters ≤3 cm, has nonetheless been critically implicated in lymph node metastasis. In the future, if accurate pathological subtypes can be determined before or during surgery, it may be possible to provide better treatment options for these patients and to provide guidance such that surgeons can develop more personalized surgical approaches. With regard to lymph node dissection, the current NCCN guidelines state that patients with NSCLC undergoing radical resection of lung cancer should also undergo systematic lymphadenectomy whenever possible and that lymph nodes N1 and N2 should be sampled during anatomical segmentectomy, even if the pathologic subtype is AIS or MIA.^[27]^ Therefore, to evaluate the relationship between pathologic subtype and lymph node metastasis, patients with lung adenocarcinoma who underwent wedge resection, lymph node sampling, or dissection of fewer than 10 nodes were excluded from this study.

The 2011 IASLC/ATS/ERS classification of lung adenocarcinoma pathologic subtype and related studies showed that AAH/AIS is characterized by pure ground glass opacity and MIA is characterized by mixed ground glass opacity on imaging.^[28–30]^ These lesions are characterized by no metastasis or recurrence, and when these 2 types of lung cancer were completely resected and the draining lymph nodes sampled, their 5-year survival rates were 100% (AIS) or nearly 100% (MIA).

Chen et al studied 803 patients with peripheral lung adenocarcinomas of diameter ≤3 cm and at clinical stage I who underwent subtotal lobectomy and a rapid frozen pathology diagnosis, which was classified as AIS, MIA, or IA.^[31]^ The results showed that for AIS and MIA, the accuracy of a rapid frozen pathology diagnosis, compared with final pathology, was 95.9%. For these patients, subfoliarlobectomy (primary segment) was performed at the margin of the lesion, and both the 5-year recurrence-free survival and 5-year OS were 100%. They also found that the larger the tumor diameter, the higher the diagnostic accuracy.

Our results also show a significant difference in the distribution of pathologic subtype as the diameter of the tumor changes. There were significant increases in the prevalences of ACI, SOL, and MIP as the diameter of the tumor increased, whereas the prevalences of AIS and MIA decreased significantly. The mechanisms underlying the association between higher tumor diameter and greater invasiveness of the pathologic subtypes remain to be determined. However, we hypothesize that less invasive tumors grow more slowly and remain dormant for longer period. We also found clear associations between VIP, serum CEA concentration, and lymph node metastasis. Tomita et al^[32]^ showed that NSCLC patients with high serum CEA had poor 5-year disease-specific survival rates than patients with normal levels and that CEA was an independent prognostic factor, suggesting that VIP and high serum CEA are associated with higher tumor aggressiveness. We also found that sex, age (>60 yrs vs ≤60 yrs), a history of smoking, and tumor location did not affect the prevalence of lymph node metastasis (*P* > .05). Women were more likely to have lung adenocarcinoma, but most had never been smokers, which is consistent with the findings of previous studies. Indeed, women are more likely to develop lung adenocarcinoma than men, and the cancer develops younger in people with no history of smoking, especially in women.^[33]^ Lan et al^[34]^ identified 3 new genetic loci and confirmed 3 previously reported genetic loci, which provides evidence of a genetic basis for lung cancer susceptibility in Asian women who have never smoked.

We should discuss 2 principal limitations of this study. First, the number of participants enrolled was relatively low, resulting in a small sample size for some of the groups, which may have affected the accuracy of the results. For example, there were only 2 participants with AIS and only 4 with MIP. Second, the study lacked clinical efficacy data and was mainly based on prognostic indicators, such as survival rate and recurrence rate. This can be explained by the postoperative follow-up period being too short to obtain meaningful data; however, we have continued to follow up these patients and will publish the longer-term outcomes in due course.

In conclusion, the pathologic subtype of lung adenocarcinomas of diameter ≤3 cm is associated with the likelihood of lymph node metastasis. Of these, AIS and MIA are not associated with lymph node metastasis; therefore, systematic lymph node dissection can be avoided. LEP tumors are associated with a low prevalence of lymph node metastasis, and it is advisable to perform rapid pathologic assessment of lymph nodes to determine whether systematic lymph node dissection is required. Other pathologic subtypes, especially SOL and MIP, are associated with a high prevalence of lymph node metastasis and require systematic lymph node dissection to achieve complete cure.

## Author contributions

**Data curation:** Wenwei Lin, Mingcheng Huang, Tianci Chai, Sui Chen.

**Formal analysis:** Wenwei Lin, Jiangbo Lin, Mingqiang Kang.

**Funding acquisition:** Mingqiang Kang.

**Investigation:** Wenwei Lin, Jiangbo Lin.

**Methodology:** Lei Gao.

**Software:** Mingcheng Huang, Zhenyang Zhang.

**Visualization:** Mingqiang Kang.

**Writing – original draft:** Wenwei Lin.
